# *aldh7a1* Regulates Eye and Limb Development in Zebrafish

**DOI:** 10.1371/journal.pone.0101782

**Published:** 2014-07-08

**Authors:** Holly E. Babcock, Sunit Dutta, Ramakrishna P. Alur, Chad Brocker, Vasilis Vasiliou, Susan Vitale, Mones Abu-Asab, Brian P. Brooks

**Affiliations:** 1 Unit on Pediatric, Developmental and Genetic Ophthalmology, Ophthalmic Genetics and Visual Function Branch, National Eye Institute, National Institutes of Health, Bethesda, Maryland, United States of America; 2 Molecular Toxicology and Environmental Health Sciences Program, Department of Pharmaceutical Sciences, University of Colorado Denver, Aurora, Colorado, United States of America; 3 Division of Biostatistic & Epidemiology, Clinical Trials Branch, National Eye Institute, National Institutes of Health, Bethesda, Maryland, United States of America; 4 Immunopathology Section, National Eye Institute, National Institutes of Health, Bethesda, Maryland, United States of America; Texas A&M University, United States of America

## Abstract

Uveal coloboma is a potentially blinding congenital ocular malformation caused by failure of the optic fissure to close during development. Although mutations in numerous genes have been described, these account for a minority of cases, complicating molecular diagnosis and genetic counseling. Here we describe a key role of *aldh7a1* as a gene necessary for normal eye development. We show that morpholino knockdown of *aldh7a1* in zebrafish causes uveal coloboma and misregulation of *nlz1*, another known contributor to the coloboma phenotype, as well as skeletal abnormalities. Knockdown of *aldh7a1* leads to reduced cell proliferation in the optic cup of zebrafish, delaying the approximation of the edges of the optic fissure. The *aldh7a1* morphant phenotype is partially rescued by co-injection of *nlz1* mRNA suggesting that *nlz1* is functionally downstream of *aldh7a1* in regulating cell proliferation in the optic cup. These results support a role of *aldh7a1* in ocular development and skeletal abnormalities in zebrafish.

## Introduction

Development of the mammalian eye begins with an evagination of forebrain neuroepithelium (the optic vesicle), which undergoes subsequent invagination to form a dual-layered optic cup. This invagination is asymmetric, such that a ventral opening (the optic fissure) forms around the fifth week of human gestation. To continue normal eye development, the two edges of the fissure must come into proximity and fuse. If the process of optic fissure closure is faulty, a uveal coloboma—a potentially blinding congenital ocular malformation—results [1], [2]. This condition can affect the iris, retina, choroid, retinal pigment epithelium (RPE), and/or the optic nerve. Worldwide, uveal coloboma affects between 0.5 and 2.6 per 10,000 births [3]–[5]. Although patients display a wide range of visual acuities, uveal coloboma may account for as much as 10% of childhood blindness [6]. The genetics of coloboma are not fully understood. While most cases are sporadic, pedigrees displaying autosomal dominant, autosomal recessive, and X-linked inheritance have been reported [7]–[10]. Coloboma patients have displayed mutations in more than twenty developmentally-regulated genes (reviewed in [11]). These genes, however, account for a minority of patients, implying that other genes may be involved in the pathogenesis of coloboma and that uveal coloboma is a complex trait involving the action of many genes in concert [10], [12], [13].

Aldehyde dehydrogenase (ALDH) family members are important for eye development. In general, members of the aldehyde dehydrogenase superfamily catalyze the oxidation of aldehydes to their corresponding carboxylic acids [14]. ALDH class 1 (ALDH1) enzymes, for instance, synthesize the important morphogen, retinoic acid (RA) and are sometimes referred to as retinal dehydrogenases (RALDH). Knockout of *Aldh1*family members in mouse models causes congenital eye malformations such as uveal coloboma/optic fissure closure defects and biallelic mutations in *ALDH1A3* in humans cause microphthalmia/anophthalmia [15]–[18].

Because of our interest in understanding the molecular mechanisms and genes involved in optic fissure closure, we investigated the expression pattern of different *Aldh/aldh* family members in publically available databases, e.g. www.Zfin.org, focusing on relevant developmental time points. Aldehyde dehydrogenase 7 family, member A1 (*aldh7a1*) is expressed strongly in the developing zebrafish eye. It is a highly evolutionarily conserved gene and is the only member of the ALDH family noted to be active in several subcellular locations—namely, the cytosol, nucleus, and mitochondria [19]. Mutations of *ALDH7A1* have been linked to the human disorder, pyridoxine-dependent epilepsy; this patient population has a broad phenotypic spectrum of severity, including some ocular findings [20]–[24]. We therefore posited that *aldh7a1*/*ALDH7A1* may play a role in ocular development.

Here we show that *aldh7a1*is dynamically expressed in ocular and skeletal tissue during zebrafish development. Knockdown of *aldh7a1* in zebrafish leads to uveal coloboma and skeletal abnormalities. In addition, we provide mechanistic evidence that these phenotypes may result from abnormal cell proliferation.

## Results

### Expression of *aldh7a1* in zebrafish during embryo development

**Figure 1:**
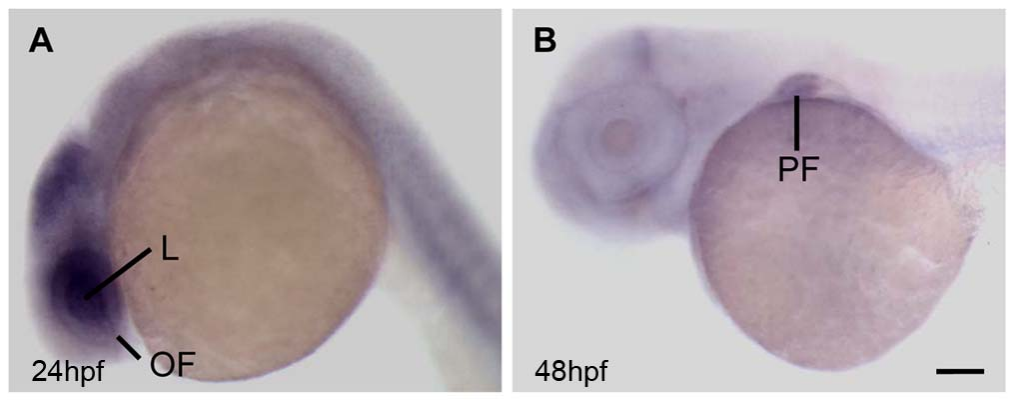
Expression pattern of *aldh7a1* in zebrafish. Whole-mount *in situ* hybridization of *aldh7a1* at (A) 24 hpf and (B) 48 hpf. L, lens; OF, optic fissure; PF, pectoral fin. Scale bar: 65 µm in A; 60 µm in B.

Whole-mount *in situ* hybridization for *aldh7a1* in zebrafish embryos was performed at approximately 9 hours post-fertilization (hpf), 18hpf, 24hpf, 36hpf, 48hpf, and 72hpf (Figure1, and Figure S1 in File S1). *aldh7a1* (GenBank: BC044367.1) was expressed ubiquitously at 18hpf (Figure S1B in File S1), but became more concentrated in the developing eye and brain by 24 hpf (Figure 1A). At 36hpf the expression was restricted to the temporal and ventral eye, cerebellum, pharyngeal arch precursors and the fin bud (Figure S1C in File S1); by 48hpf, overall expression was decreased (Figure 1B). Pectoral fin expression persisted at around 48hpf (Figure 1B) and persisted through 72hpf (Figure S1 in File S1). We therefore inferred that *aldh7a1* were likely important in the development of these structures.

**Figure 2:**
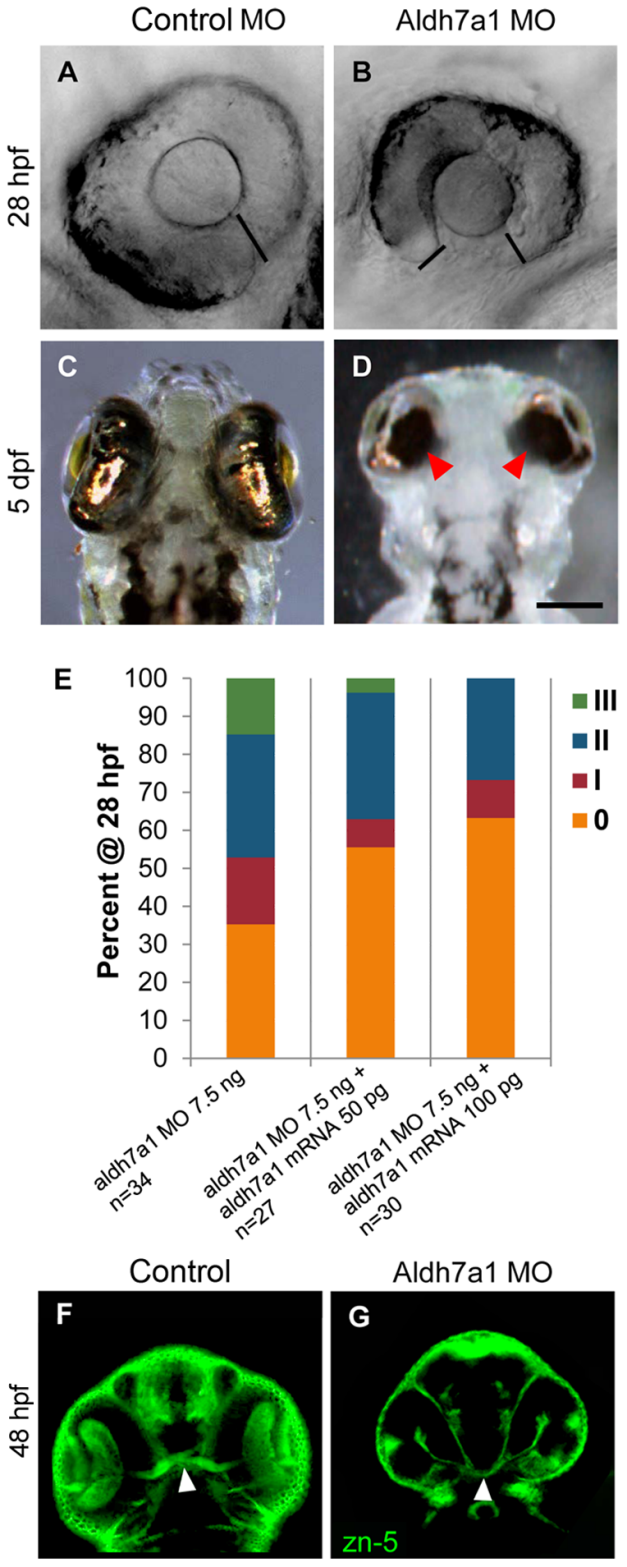
Aldh7a1 is important for optic fissure closure. (A–D) Injection of Aldh7a1 morpholino results in failure of optic fissure to close at 28 hpf and sustained at 5 dpf. (A) Eye of control morpholino (MO) injected embryo at 28 hpf, (B) Eye of *aldh7a1* morphant at 28 hpf; (C) Ventral view of eye in control MO embryo at 5 dpf, (D) Ventral view of eye in embryos injected with 7.5 ng Aldh7a1 MO at 5 dpf, black bars indicate edges of optic fissures; (E) Bar graph demonstrate distribution of eye phenotypes 0, I, II, and III at 28 hpf following 7.5 ng Aldh7a1 MO injection followed by partial rescue of phenotype upon co-injection of two doses of *aldh7a1* mRNA. All control MO injected embryos displayed “0” phenotype. (F) zn-5 staining of control MO injected embryos at 48 hpf compared to (G) *aldh7a1* morphant which displays optic nerve hypoplasia, arrow-heads indicate optic nerve. Scale bar: 65 µm in A,B; 125 µm in C,D; 75 µm in F,G.

**Figure 3:**
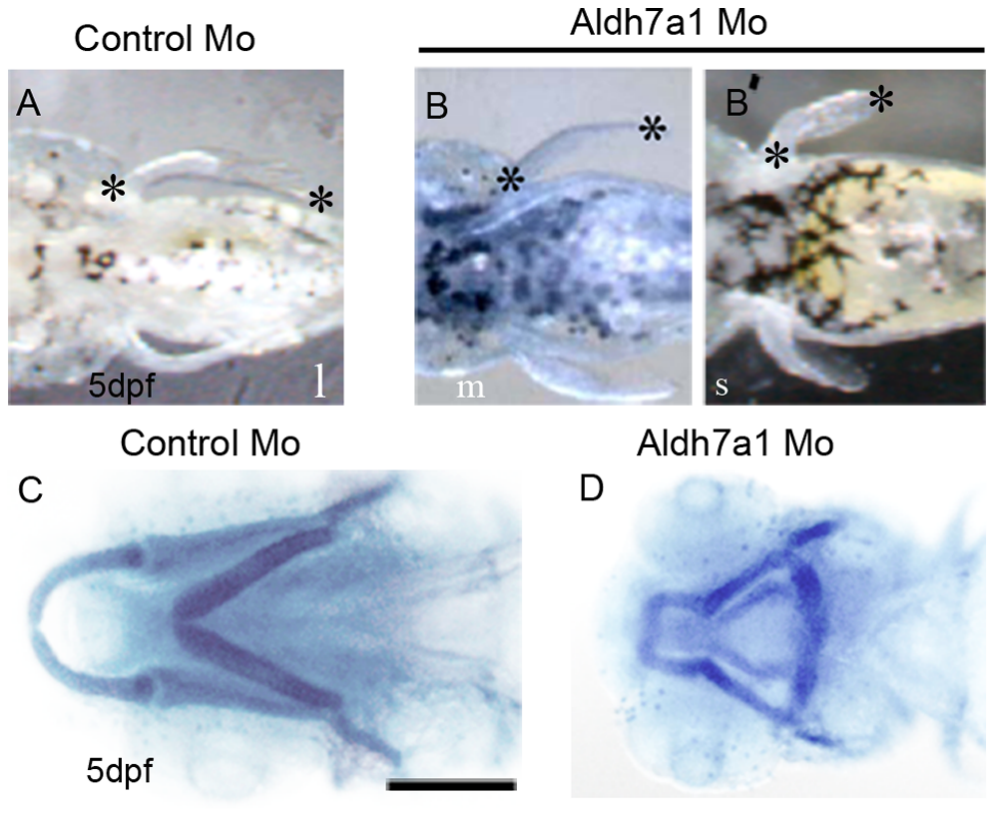
Aldh7a1 morpholino knockdown embryos show defects in pectoral fin and cartilage development. (A–B′) Fin phenotypes long (l), medium (m), and short (s) classified by length at 5 dpf, marked by black asterisks. All control MO embryos displayed “Long” fins (A) and Aldh7a1MO injected embryos develop medium (B, 6%) or short (B′, 10%) fin. (C–D) Ventral view of Alcian blue staining of jaw- cartilages in control MO (C) and *aldh7a1*morphant (D) embryos. Scale bar: 350 µm in A–B′; 125 µm in C,D.

To further investigate the role of *aldh7a1* during embryonic development, we performed reduction-of-function analysis by microinjection of morpholino-oligonucleotides (MOs) targeted to translation start sites of the corresponding mRNA. In order to do semi-quantitative analysis of eye phenotypes, we used a four-point grading scale (Figure S2 in File S1), “0” being the control phenotype where the two edges of the optic fissure fused normally. Uninjected and control-MO fish behaved identically in this and other measures used throughout this paper. Categories “I”, “II”, and “III” indicate increasing severity of coloboma phenotype where the two edges of the fissure remained increasingly further apart, similar to our previous report [25]. Knockdown of *aldh7a1* by Aldh7a1 MO1in zebrafish embryos resulted in bent tail (Figure S3A, D in File S1); coloboma of the eye evident at 28 hpf which persists until at least 5–6 days post-fertilization (dpf) (Figure 2B,D; Figure S3 E,F in File S1) compared to control embryos (Figure 2A,C; Figure S3B,C in File S1) where the optic fissure had nearly fused at 28 hpf (Figure 2A). Similar results were obtained by injecting Aldh7a1 MO2 in zebrafish embryos (data not shown). However, co-injection of Aldh7a1 MO1 and Aldh7a1 MO2 also resulted in coloboma (Figure S3 I, J in File S1), but with lower total concentrations of MO compared to controls (Figure S3 G,H in File S1). In this study most of the experiments were performed by using Aldh7a1 MO1, unless otherwise stated. Co-injection of *aldh7a1* mRNA with the Aldh7a1 MO partially rescued the morphant phenotype, defined by a greater number of “0”/mildly affected embryos and fewer severely affected embryos based on our four-point scale of optic fissure closure (Figure 2E, p = 0.03 at 100 pg mRNA). At 48hpf, staining of axons using zn-5 antibody revealed that *aldh7a1* morphant fish displayed smaller eyes and optic nerves compared to control fish (Figure 2F,G). At 5dpf, morphant fish showed shortened pectoral fins (Figure 3A,B, B′). We ascertained the cartilage structure of the control and morphant fish at 5dpf using Alcian blue staining. Fish exhibiting the more severe fin phenotypes (data not shown) also displayed abnormal cartilage structure in the head and jaw (Figure 3C, D). We could not show mRNA rescue of the fin and cartilage phenotypes because the mRNA did not persist until 5dpf.

### Knockdown of *aldh7a1* caused misregulation of genes necessary for proper eye development and was partially rescued by co-injection of *nlz1* mRNA

**Figure 4:**
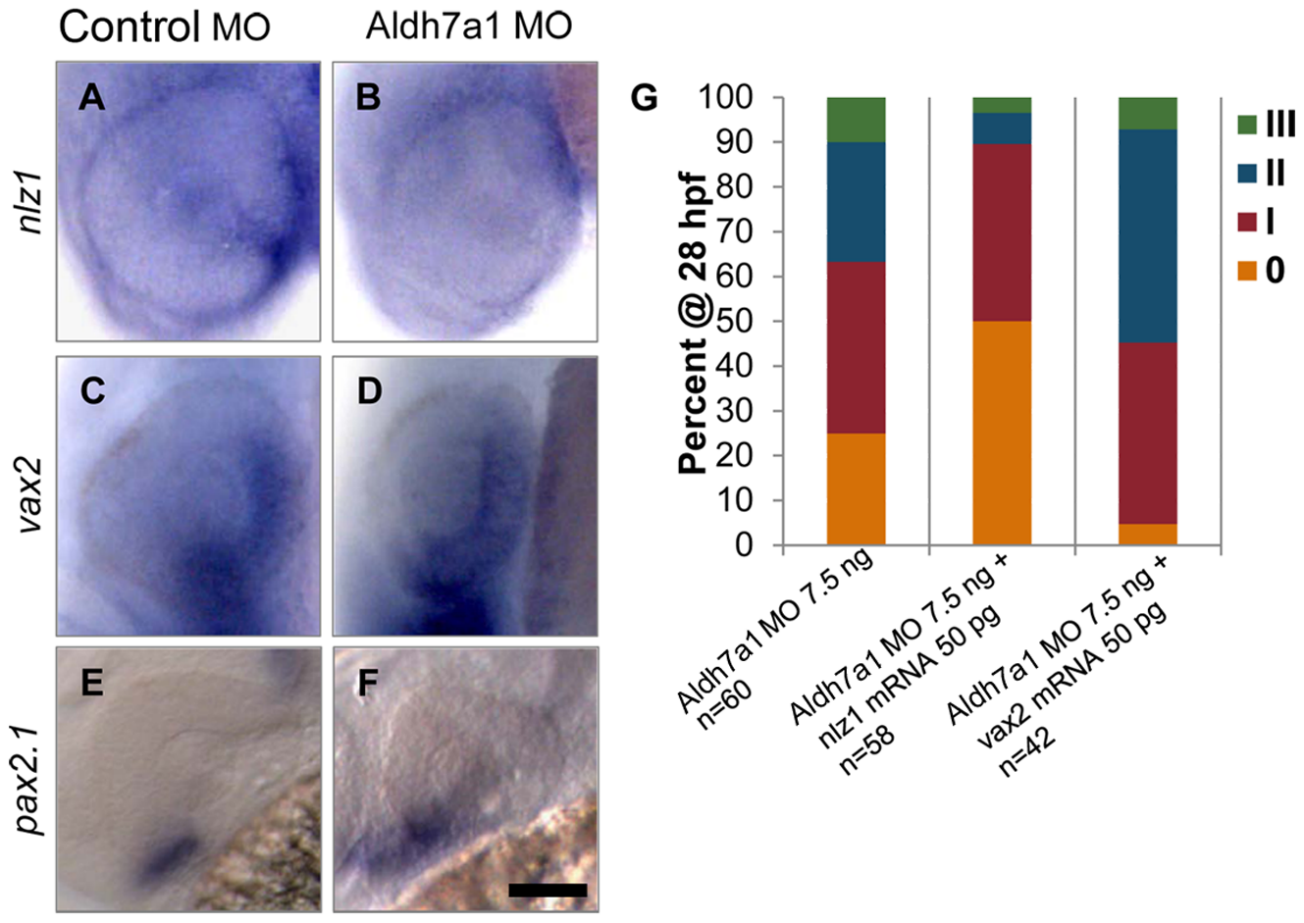
Expression pattern of genetic eye development markers in control and *aldh7a1* morphant embryos. (A) Expression of *nlz1* in optic fissure is down-regulated in (B) *aldh7a1* morphant fish. *vax2* and *pax2.1* do not seem to show significant change in expression between control MO (C,E) and *nlz1* morphant (D, F) fish. (G) Co-injection of *nlz1* mRNA resulted in partial rescue of *aldh7a1* MO phenotype, examined at 28 hpf, while co-injection of *vax2* mRNA showed no change. Scale bar: 65 µm.

Because of the strong expression of *aldh7a1* in the developing zebrafish eye and the ventral nature of uveal coloboma, we examined expression of genetic markers known to be important in eye ventralization, including *nlz1*, *vax2*, *pax2.1, nlz2*, and *vax1* in control (Figure 4A,C,E; data not shown) as well as in *aldh7a1* morphant (Figure 4B,D,F; data not shown) embryos. Of these, only *nlz1* (GenBank: AF222996.1) showed consistent down regulation in expression in *aldh7a1* morphant eyes at 24hpf. The expression of *nlz1* that was seen in the optic fissure and periocular mesenchyme in control embryos (Figure 4A) was lost in *aldh7a1* morphant embryos (Figure 4B). This loss of expression led us to hypothesize that *nlz1* functions downstream of *aldh7a1*. To test this hypothesis, we attempted to rescue the a*ldh7a1* morphant phenotype by co-injecting the Aldh7a1 MO with *nlz1* mRNA. Because of the well-established role that *vax2* plays in the ventralization of the eye during development, we performed the same rescue experiment with *vax2* mRNA as a negative control to confirm specificity. We found that, as predicted, co-injection of *vax2* mRNA did not rescue the morphant phenotype and may actually increase its severity (Figure 4G, p = 0.02); however, co-injection of *nlz1* mRNA partially rescued the *aldh7a1*morphants (p = 0.001). This, again, was demonstrated by a greater number of “0”/mildly affected embryos and fewer severely affected embryos. This partial rescue indicated that *nlz1* functions downstream of *aldh7a1*.

### Retinoic acid treatment does not rescues *aldh7a1* morphant phenotype

It is well documented that RA plays an important role in eye morphogenesis. Members of the ALDH superfamily (specifically, the ALDH1A family) oxidize retinaldehyde to RA. *Aldh1a3* ^-/-^ (*Raldh3*) knockout mice display coloboma, and although *Aldh1a1* ^-/-^ (*Raldh1*) knockout mice did not have a recognizable ophthalmic phenotype, double knockout *Aldh1a1* ^-/-^; *Alldh1a3* ^-/-^ mice displayed an even more severe eye phenotype [26]. In zebrafish, blocking RA during development resulted in coloboma [27]. Additionally, RA regulates *nlz1* in zebrafish, demonstrated by a significant decrease in *nlz1* expression in embryos where RA signaling is blocked [28]. More recently recessive mutation in *ALDH1A3* have been associated with severe micropthalmia, anophthalmia, and hypoplasia of the optic tract [17], [18]. These existing connections of RA to coloboma led us to pursue it as a chemical that is potentially involved in the *aldh7a1* pathway. We attempted to rescue the morphant phenotype with one- or two-hour incubations in varying concentrations of RA at the 2 somite stage, the 10 somite stage and the 20-somite stage, when eye development is dynamic. We quantified the number of embryos displaying each of the four optic fissure grades at 28 hpf and found that RA-treated morphants did not differ from control, untreated morphants (Figure S4 in File S1). RA concentrations beyond 3 µM lead to deformed and dead embryos at 28hpf (data not shown). Thus we conclude that exogenous RA is not able to rescue the *aldh7a1* morphant eye phenotype and is less likely to be the endogenous substrate.

### Knockdown of *aldh7a1* alters cell proliferation in developing eye

**Figure 5:**
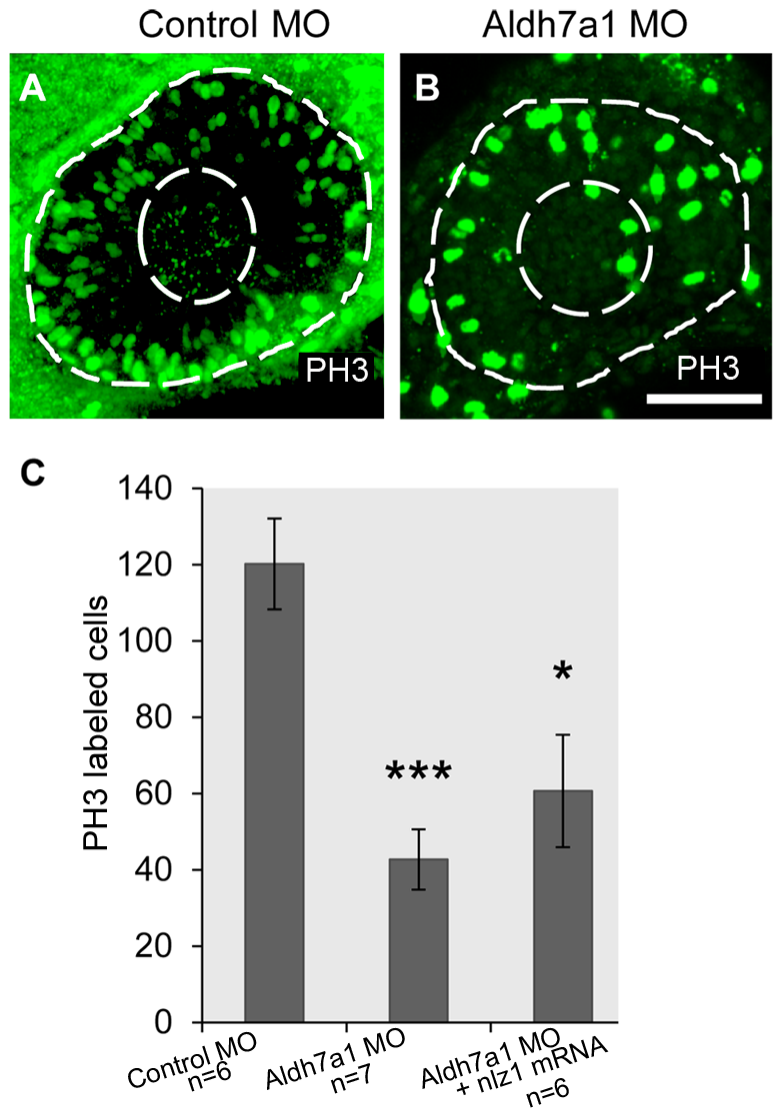
Aldh7a1 is required for retinal cell proliferation. (A–B) Dividing cells in developing eye were labeled with phosophohistone-3 antibody (H3P) in (A) Control MO and (B) *alh7a1*morphant embryos at 28 hpf. (A) and (B) are projection images of z-stacks through the depth of the eye. (C) Average number of dividing cells per eye quantified for control MO (n = 6), Nlz1 MO (n = 7), *nlz1* mRNA (n = 6) rescued Statistical significance indicated above columns *P<0.05, **P<0.01, ***P<0.0001. Scale bar: 65 µm.

A prerequisite for optic fissure closure is the approximation of its two edges at the appropriate time during development. We noted that the edges of the optic fissure were widely spaced in our morphant fish, and hypothesized that this may be due to a decrease in the rate of cell division required to acquire a normal optic fissure configuration. We compared the number of dividing cells in the retina between uninjected control and morphant embryos at 24 hpf using an anti-phosphohistone H3 (PH3) antibody to label active histones in the M phase of the cell cycle. Qualitatively, there is a clear reduction in labeled cells in the morphant eye (Figure 5B) compared to control eye (Figure 5A). When quantified, the average number of labeled cells in morphant eyes compared to control eyes was statistically lower (Figure 5C). We also labeled dividing cells in the eyes of morphants rescued with *nlz1* mRNA. The eyes of rescued morphants showed a statistically significant recovery in the number of PH3 labeled cells (Figure 5C). In addition to counting total number of PH3 positive cells in the eye, we have analyzed total number of PH3 positive cells per 1000 µm^2^ areas in the eye of control MO (5.2 cells/1000 µm^2^) injected embryos, *aldh7a1* morphants (2 cells/1000 µm^2^) and rescued morphants (4 cells/1000 µm^2^) embryos. To address the morpholino mediated nonspecific effects on apoptotic cell death in the eye, we performed immunostaining by using anti-active caspase 3 antibody in control MO and *aldh7a1* morphant embryos; no significant difference in the staining was observed (Figure S5 in File S1). These findings offer plausible evidence that uveal coloboma involves an *aldh7a1*-dependent mechanism that disrupts the cell proliferation.

## Discussion

In this study, our aim was to understand function of *aldh7a1* during embryo development. *ALDH7A1* is part of a superfamily of aldehyde dehydrogenases involved in the NAD(P)^+^-dependent oxidation of reactive aldehydes to carboxylic acids, as well as the metabolism of other important molecules such as tetrahydrofolate, γ-aminobutyric acid, RA, and betaine [14]. The gene is also suspected to play a role in regulation of hyperosmotic/oxidative stress and the cell cycle [19], [29], [30]. As previously noted, ALDH7A1 is unique amongst this family in that it is localized to the cytosol, nucleus, and mitochondria and is highly conserved through evolution, implying one or more essential roles in cell physiology [19]. In fact, ALDH7A1 was originally named “antiquitin” referring to its ancient origins and the high level of amino acid similarity of the homologues.

Biallelic mutations in *ALDH7A1* have been associated with pyridoxine-dependent epilepsy (PDE) and folinic acid responsive seizures in humans [21], [23]. Although we were not equipped to evaluate seizure activity in our morphants, many of PDE patients also have developmental abnormalities of the CNS, including optic nerve hypoplasia—a phenotype observed in our zebrafish model [20]. Although uveal coloboma and skeletal abnormalities have not been reported in PDE, it is unclear from the literature whether these phenotypes were systematically ascertained in the patients reported. An informative follow-up to this study would be the careful examination of patients with PDE for subtle ocular and skeletal abnormalities consistent with the zebrafish phenotype. Since *ALDH7A1* is a susceptibility gene for osteoporosis and is believed to play a role in bone formation and maintenance [31] as well as cancer metastasis[32], assessing these specific phenotypes in PDE patients (and, perhaps, their carrier parents) would be of particular interest. While published data do not support a clear link between ALDH7A1 mutations and optic fissure closure *per se*, they do not exclude the possibility that regulation of *ALDH7A1* is an important modifier of ocular development.

Based on basic developmental principles, our lab and others have posited that in order for the optic fissure to close, the two edges of the optic cup must approximate in the correct anatomic location, at the correct developmental stage, and express the correct complement of genes; any event that disrupts one or more of these conditions results in uveal coloboma [1], [2]. Abnormal regulation of the cell cycle would therefore be predicted to disrupt ocular morphogenesis by interfering with this developmental program. Other zebrafish models of coloboma, such as knockdown of *chd7* and *gdf6*, in fact, result in reduced cell proliferation in the developing eye [33], [34]. ALDH7A1 has been shown to localize, in part, to the nucleus and to be up-regulated using the G(1)-S phase transition [30]. Our observation that knockdown of *aldh7a1* results in reduced cell proliferation is consistent with these findings. Furthermore, the observation that *nlz1/NLZ1* which is important in optic fissure closure [25] acts functionally downstream of *aldh7a1* is also consistent with the observation that *NLZ1* is important in tumor cell division and metastasis [35]. The precise mechanism by which *aldh7a1* levels affect *nlz1* activity is an ongoing area of investigation in our laboratory. Our attempts to rescue the morphant phenotype with *vax2* mRNA may make the morphant phenotype more pronounced, consistent with the observations of Liu *et al.* that *Xvax2* mRNA (the *Xenopus* ortholog of *vax2*) is an inhibitor of cell proliferation in the developing eye and brain [36].

Several proteins in the ALDH superfamily have the primary function of converting retinal to RA (e.g., ALDH1A1, ALDH1A2, ALDH1A3) [14]. Coloboma results when zebrafish embryos were treated with citral, a compound that decreases the production of RA in the ventral retina. This phenotype was partially rescued by incubation in RA [27]. When retinoic acid receptor (RAR) signaling was inhibited by treating zebrafish embryos with AGN194310 (AGN), coloboma was present in 75% of embryos quantified at 60 hpf [28]. Our experience with *aldh7a1* revealed no relationship with RA, in that incubation of morphants in various RA concentrations showed no rescue of phenotype. Consistent with this observation, Tang et al. found that Aldh7a1 from the seabream fish—which shares 84% identity with ALDH7A1—could not utilize retinal as a substrate [37]. Lastly, *in silico* analysis demonstrates that ALDH7A1 has low sequence identity with other, known RA-metabolizing members of the superfamilmy: ALDH1A1 (25.7%), ALDH7A2 (28%), and ALDH1A3 (25.7%). Taken together, these observations make it unlikely that Aldh7a1 plays a major role in RA production in our model system.

In summary, we demonstrate that *aldh7a1* has a critical role in eye and skeletal development in zebrafish. The optic fissure closure defects in our morphant fish occurred at least in part because of reduced *nlz1* expression and reduced cell proliferation. Regulation of *ALDH7A1/*ALDH7A1 expression and/or activity may therefore play an important role in human optic fissure closure, optic nerve formation and skeletal development.

## Materials and Methods

### Ethics Statement

This study was carried out in strict accordance with the recommendations in the Guide for the Care and Use of Laboratory Animals of the National Institutes of Health. All animal experiments were conducted under protocols approved by the National Eye Institute's Animal Care and Use Committee (NEI ACUC) at the National Institutes of Health (ASP#NEI-648).

### Fish Husbandry

AB×TL strains of zebrafish were raised and maintained according to standard protocols [38]. Embryos were collected after natural spawning and maintained at 28.5°C.

### RNA Probe Synthesis and Whole-Mount *In Situ* Hybridization

Antisense RNA probes were synthesized from full-length cDNA IMAGE clones (Open Biosystems, Huntsville, AL) using digoxigenin RNA-labelling kit (Roche, Indianapolis, IN). Whole-mount *in situ* hybridization was carried out as described [39] at 65°C using probes for *aldh7a1, nlz1* [25], *nlz2* [25], *vax1* [40], *vax2* [40], and *pax2.1* [41].

### Morpholino Gene Knockdown and mRNA Rescue in Zebrafish

Translation-blocking morpholino oligonucleotides were obtained from Gene Tools, LLC (Philomath, OR), and diluted in 0.1 M KCl. Zebrafish embryos were injected with 7.5 ng Aldh7a1 MO1, (5′TCGGACACTCGGCAACAGTTTATGC3′), 10 ng of Aldh7a1 MO2 (5'AGTCGCGCAAGTC TCAGCGTCAGCA3′) and 10 ng of control MO (5'ATCCAGGAGGCAGTTCGCTCATCTG3') at the 1–2 cell stage. In some experiments, the embryos were co-injected with 2 ng Aldh7a1 MO1 and 2 ng Aldh7a1 MO2 (4 ng, a lower total concentration of MO). The ORFs for *aldh7a1*, *nlz1*, and *vax2* were amplified by PCR from IMAGE clones obtained from Open Biosystems (Table S1). PCR products were purified and sub-cloned into pCS2^+^ [42]. Recombinant clones were linearized with NotI and capped mRNA were synthesized *in vitro* using mMESSAGE mMACHINE kit (Ambion, Grand Island, NY). Zebrafish embryos were co-injected with 50–100 pg of synthetic mRNA and 7.5 ng of Aldh7a1 MO at the 1–2 cell stage. In rescue experiments, datasets were compared using Chi-squared analysis. Because some severity scores contained a low number of embryos, affected phenotypes were sometimes grouped together for analysis (e.g., grades II and III were grouped in the analysis of *nlz1* and *vax2* rescue of *alhd7a1* morphants.)

### Alcian Blue Staining

Cartilage was stained with Alcian Blue according to a protocol modified from Neuhauss *et al*. (1996) [43]. Embryos at 5dpf were fixed overnight in 4% PFA and then washed in PBST. The embryos were bleached of pigments in 1 mL of solution containing 100 µL 30% H_2_O_2_, 100 µL 1% KOH, 800 µL ddH_2_O. After rinsing again with PBST, they were stained overnight in 0.1% Alcian Blue dissolved in acidic ethanol (70% EtOH, 5% concentrated HCl). Post-staining, embryos were washed extensively in acidic ethanol and then dehydrated and transferred to 80% glycerol for storage and visualization.

### Retinoic Acid treatment

Developing control and morphant zebrafish embryos were incubated in various concentrations of retinoic acid prepared according to Hyatt *et al.* (1992) [44] for 1 hour at the 20-somite stage, and 2 hour at the 2- somite stage, and 10-somite stage. The embryos were washes several time in embryo media after RA treatment, and incubated in embryo media until 28hpf.

### Immunohistochemistry

Embryos were fixed overnight in 4% PFA and washed with PBT0.2 (1X PBS with 0.2% Triton X-100). Blocking was completed for 30 minutes at RT in solution of PBT0.2 with 2% goat serum. Proliferating cells were labeled with rabbit anti-phosphohistone-H3 (H3P, Millipore, Billerica, MA) primary antibody at 1∶250 and incubated overnight. Embryos were rinsed thoroughly with PBT0.2 and then incubated for 2 hours at RT with secondary antibody Alexa Fluor 488 rabbit anti-mouse IgG (Molecular Probes, Grand Island, NY) at 1∶400 for visualization by fluorescence microscopy. The same procedure was used to analyze optic nerve with zn-5 primary antibody to label axons. Mouse-conjugated zn-5 (ZIRC 021009) was diluted at 1∶200 and embryos were incubated overnight. Alexa Fluor 488 goat anti-mouse IgG (Molecular Probes, Grand Island, NY) at 1∶400 was used as secondary antibody. Apoptotic cells were labeled with rabbit anti- active- caspase3 antibody (BD Pharmingen), and Alexa Fluor 568 (Molecular Probes, Grand Island, NY) goat anti-mouse IgG was used as secondary antibody

### Imaging

Confocal imaging was performed using a Zeiss LSM 700 microscope with ZEN Software (Carl Zeiss Microscopy, LLC, Thornwood, NY). Z-stacks were analyzed using Velocity Image Analysis software. Additional micrographs were taken with a Leica M205 FA stereo microscope using Leica Application Suite V3 (Leica Microsystems, Inc., Buffalo Grove, IL).

## Supporting Information

File S1 **Supporting figures and table.** **Figure S1**, *aldh7a1* expression. **Figure S2**, Grades of severity for eye development. **Figure S3**, *aldh7a1* loss-of-function phenotype. **Figure S4**, *aldh7a1* morphant phenotype is not rescued by retinoic acid. **Figure S5**, Coloboma in *aldh7a1*morphant fish is not due to apoptosis. **Table S1**, List of Image clones and Oligonucleotides used in this study.
(PDF)

## References

[1] Chang L, Blain D, Bertuzzi S, Brooks BP (2006) Uveal coloboma: clinical and basic science update. Curr Opin Ophthalmol 17: 447–470. 16932062 10.1097/01.icu.0000243020.82380.f6

[2] Gregory-Evans CY, Williams MJ, Halford S, Gregory-Evans K (2004) Ocular coloboma: a reassessment in the age of molecular neuroscience. J Med Genet 41: 881–891. 15591273 10.1136/jmg.2004.025494 PMC1735648

[3] Bermejo E, Martinez-Frias ML (1998) Congenital eye malformations: clinical-epidemiological analysis of 1,124,654 consecutive births in Spain. Am J Med Genet 75: 497–504. 9489793

[4] Hornby SJ, Gilbert CE, Rahi JK, Sil AK, Xiao Y, et al (2000) Regional variation in blindness in children due to microphthalmos, anophthalmos and coloboma. Ophthalmic Epidemiol 7: 127–138. 10934463

[5] Nakamura KM, Diehl NN, Mohney BG (2011) Incidence, ocular findings, and systemic associations of ocular coloboma: a population-based study. Arch Ophthalmol 129: 69–74. 21220631 10.1001/archophthalmol.2010.320 PMC3126628

[6] Maumenee IH, Mitchell TN (1990) Colobomatous malformations of the eye. Trans Am Ophthalmol Soc 88: 123–132 discussion 133–125. 2095017 PMC1298581

[7] Zlotogora J, Legum C, Raz J, Merin S, BenEzra D (1994) Autosomal recessive colobomatous microphthalmia. Am J Med Genet 49: 261–262. 8209881 10.1002/ajmg.1320490302

[8] Pagon RA, Kalina RE, Lechner DJ (1981) Possible autosomal-recessive ocular coloboma. Am J Med Genet 9: 189–193. 7282780 10.1002/ajmg.1320090304

[9] Lehman DM, Sponsel WE, Stratton RF, Mensah J, Macdonald JC, et al (2001) Genetic mapping of a novel X-linked recessive colobomatous microphthalmia. Am J Med Genet 101: 114–119. 11391653 10.1002/ajmg.1330

[10] Morrison D, FitzPatrick D, Hanson I, Williamson K, van Heyningen V, et al (2002) National study of microphthalmia, anophthalmia, and coloboma (MAC) in Scotland: investigation of genetic aetiology. J Med Genet 39: 16–22. 11826019 10.1136/jmg.39.1.16 PMC1734963

[11] Brooks BP (2013) Anophthalmia, Microphthalmia and Uveal Coloboma. In: Rimoin DL, Pyeritz RE, Korf BR, editors. Emery and Rimoin's Principles and Practice of Medical Genetics. 6th edition ed: Academic Press. pp. 3952–3966.

[12] Gonzalez-Rodriguez J, Pelcastre EL, Tovilla-Canales JL, Garcia-Ortiz JE, Amato-Almanza M, et al (2010) Mutational screening of CHX10, GDF6, OTX2, RAX and SOX2 genes in 50 unrelated microphthalmia-anophthalmia-coloboma (MAC) spectrum cases. Br J Ophthalmol 94: 1100–1104. 20494911 10.1136/bjo.2009.173500

[13] Zhang X, Li S, Xiao X, Jia X, Wang P, et al (2009) Mutational screening of 10 genes in Chinese patients with microphthalmia and/or coloboma. Mol Vis 15: 2911–2918. 20057906 PMC2802294

[14] Marchitti SA, Brocker C, Stagos D, Vasiliou V (2008) Non-P450 aldehyde oxidizing enzymes: the aldehyde dehydrogenase superfamily. Expert Opin Drug Metab Toxicol 4: 697–720. 18611112 10.1517/17425250802102627 PMC2658643

[15] Matt N, Dupe V, Garnier JM, Dennefeld C, Chambon P, et al (2005) Retinoic acid-dependent eye morphogenesis is orchestrated by neural crest cells. Development 132: 4789–4800. 16207763 10.1242/dev.02031

[16] Dupe V, Matt N, Garnier JM, Chambon P, Mark M, et al (2003) A newborn lethal defect due to inactivation of retinaldehyde dehydrogenase type 3 is prevented by maternal retinoic acid treatment. Proceedings of the National Academy of Sciences of the United States of America 100: 14036–14041. 14623956 10.1073/pnas.2336223100 PMC283541

[17] Fares-Taie L, Gerber S, Chassaing N, Clayton-Smith J, Hanein S, et al (2013) ALDH1A3 mutations cause recessive anophthalmia and microphthalmia. Am J Hum Genet 92: 265–270. 23312594 10.1016/j.ajhg.2012.12.003 PMC3567280

[18] Yahyavi M, Abouzeid H, Gawdat G, de Preux AS, Xiao T, et al (2013) ALDH1A3 loss of function causes bilateral anophthalmia/microphthalmia and hypoplasia of the optic nerve and optic chiasm. Hum Mol Genet 22: 3250–3258. 23591992 10.1093/hmg/ddt179 PMC3723310

[19] Brocker C, Lassen N, Estey T, Pappa A, Cantore M, et al (2010) Aldehyde dehydrogenase 7A1 (ALDH7A1) is a novel enzyme involved in cellular defense against hyperosmotic stress. J Biol Chem 285: 18452–18463. 20207735 10.1074/jbc.M109.077925 PMC2881771

[20] Mills PB, Footitt EJ, Mills KA, Tuschl K, Aylett S, et al (2010) Genotypic and phenotypic spectrum of pyridoxine-dependent epilepsy (ALDH7A1 deficiency). Brain: a journal of neurology 133: 2148–2159. 20554659 10.1093/brain/awq143 PMC2892945

[21] Mills PB, Struys E, Jakobs C, Plecko B, Baxter P, et al (2006) Mutations in antiquitin in individuals with pyridoxine-dependent seizures. Nat Med 12: 307–309. 16491085 10.1038/nm1366

[22] Bennett CL, Chen Y, Hahn S, Glass IA, Gospe SM Jr (2009) Prevalence of ALDH7A1 mutations in 18 North American pyridoxine-dependent seizure (PDS) patients. Epilepsia 50: 1167–1175. 19128417 10.1111/j.1528-1167.2008.01816.x

[23] Gallagher RC, Van Hove JL, Scharer G, Hyland K, Plecko B, et al (2009) Folinic acid-responsive seizures are identical to pyridoxine-dependent epilepsy. Ann Neurol 65: 550–556. 19142996 10.1002/ana.21568

[24] Scharer G, Brocker C, Vasiliou V, Creadon-Swindell G, Gallagher RC, et al (2010) The genotypic and phenotypic spectrum of pyridoxine-dependent epilepsy due to mutations in ALDH7A1. J Inherit Metab Dis 33: 571–581. 20814824 10.1007/s10545-010-9187-2 PMC3112356

[25] Brown JD, Dutta S, Bharti K, Bonner RF, Munson PJ, et al (2009) Expression profiling during ocular development identifies 2 Nlz genes with a critical role in optic fissure closure. Proc Natl Acad Sci U S A 106: 1462–1467. 19171890 10.1073/pnas.0812017106 PMC2631080

[26] Molotkov A, Molotkova N, Duester G (2006) Retinoic acid guides eye morphogenetic movements via paracrine signaling but is unnecessary for retinal dorsoventral patterning. Development 133: 1901–1910. 16611695 10.1242/dev.02328 PMC2833011

[27] Marsh-Armstrong N, McCaffery P, Gilbert W, Dowling JE, Drager UC (1994) Retinoic acid is necessary for development of the ventral retina in zebrafish. Proc Natl Acad Sci U S A 91: 7286–7290. 8041782 10.1073/pnas.91.15.7286 PMC44384

[28] Lupo G, Gestri G, O'Brien M, Denton RM, Chandraratna RA, et al (2011) Retinoic acid receptor signaling regulates choroid fissure closure through independent mechanisms in the ventral optic cup and periocular mesenchyme. Proc Natl Acad Sci U S A 108: 8698–8703. 21555593 10.1073/pnas.1103802108 PMC3102374

[29] Brocker C, Cantore M, Failli P, Vasiliou V (2011) Aldehyde dehydrogenase 7A1 (ALDH7A1) attenuates reactive aldehyde and oxidative stress induced cytotoxicity. Chem Biol Interact 191: 269–277. 21338592 10.1016/j.cbi.2011.02.016 PMC3387551

[30] Chan CL, Wong JW, Wong CP, Chan MK, Fong WP (2011) Human antiquitin: structural and functional studies. Chem Biol Interact 191: 165–170. 21185811 10.1016/j.cbi.2010.12.019

[31] Guo Y, Tan LJ, Lei SF, Yang TL, Chen XD, et al (2010) Genome-wide association study identifies ALDH7A1 as a novel susceptibility gene for osteoporosis. PLoS Genet 6: e1000806. 20072603 10.1371/journal.pgen.1000806 PMC2794362

[32] van den Hoogen C, van der Horst G, Cheung H, Buijs JT, Pelger RC, et al (2011) The aldehyde dehydrogenase enzyme 7A1 is functionally involved in prostate cancer bone metastasis. Clin Exp Metastasis 28: 615–625. 21647815 10.1007/s10585-011-9395-7 PMC3198191

[33] Balow SA, Pierce LX, Zentner GE, Conrad PA, Davis S, et al (2013) Knockdown of fbxl10/kdm2bb rescues chd7 morphant phenotype in a zebrafish model of CHARGE syndrome. Developmental Biology 382: 57–69. 23920116 10.1016/j.ydbio.2013.07.026 PMC3816111

[34] French CR, Stach TR, March LD, Lehmann OJ, Waskiewicz AJ (2013) Apoptotic and proliferative defects characterize ocular development in a microphthalmic BMP model. Invest Ophthalmol Vis Sci 54: 4636–4647. 23737474 10.1167/iovs.13-11674

[35] Slorach EM, Chou J, Werb Z (2011) Zeppo1 is a novel metastasis promoter that represses E-cadherin expression and regulates p120-catenin isoform expression and localization. Genes Dev 25: 471–484. 21317240 10.1101/gad.1998111 PMC3049288

[36] Liu M, Liu Y, Lupo G, Lan L, Barsacchi G, et al (2008) A role for Xvax2 in controlling proliferation of Xenopus ventral eye and brain progenitors. Dev Dyn 237: 3387–3393. 18942138 10.1002/dvdy.21763

[37] Tang WK, Chan CB, Cheng CH, Fong WP (2005) Seabream antiquitin: molecular cloning, tissue distribution, subcellular localization and functional expression. FEBS Lett 579: 3759–3764. 15967446 10.1016/j.febslet.2005.05.070

[38] Westerfield M (2000) The zebrafish book; A Guide for the Laboratory Use of Zebrafish (Danio rerio). Eugene,OR: University of Oregon Press.

[39] Thisse C, Thisse B (2008) High-resolution in situ hybridization to whole-mount zebrafish embryos. Nat Protoc 3: 59–69. 18193022 10.1038/nprot.2007.514

[40] Take-uchi M, Clarke JD, Wilson SW (2003) Hedgehog signalling maintains the optic stalk-retinal interface through the regulation of Vax gene activity. Development 130: 955–968. 12538521 10.1242/dev.00305

[41] Krauss S, Johansen T, Korzh V, Fjose A (1991) Expression of the zebrafish paired box gene pax[zf-b] during early neurogenesis. Development 113: 1193–1206. 1811936 10.1242/dev.113.4.1193

[42] Turner DL, Weintraub H (1994) Expression of achaete-scute homolog 3 in Xenopus embryos converts ectodermal cells to a neural fate. Genes Dev 8: 1434–1447. 7926743 10.1101/gad.8.12.1434

[43] Neuhauss SC, Solnica-Krezel L, Schier AF, Zwartkruis F, Stemple DL, et al (1996) Mutations affecting craniofacial development in zebrafish. Development 123: 357–367. 9007255 10.1242/dev.123.1.357

[44] Hyatt GA, Schmitt EA, Marsh-Armstrong NR, Dowling JE (1992) Retinoic acid-induced duplication of the zebrafish retina. Proc Natl Acad Sci U S A 89: 8293–8297. 1518861 10.1073/pnas.89.17.8293 PMC49904

