# Supplementary material for: aldh7a1 Regulates Eye and Limb Development in Zebrafish
Source: PLoS One. 2014 Jul 8;9(7):e101782. doi: 10.1371/journal.pone.0101782 (PMC4086958; doi:10.1371/journal.pone.0101782)
Supplement: File S1 — Supporting figures and table. Figure S1, aldh7a1 expression. Figure S2, Grades of severity for eye development. Figure S3, aldh7a1 loss-of-function phenotype. Figure S4, aldh7a1 morphant phenotype is not rescued by retinoic acid. Figure S5, Coloboma in aldh7a1morphant fish is not due to apoptosis. Table S1, List of Image clones and Oligonucleotides used in this study. (PDF) [file pone.0101782.s001.pdf]

Supplementary Data

Figure S1

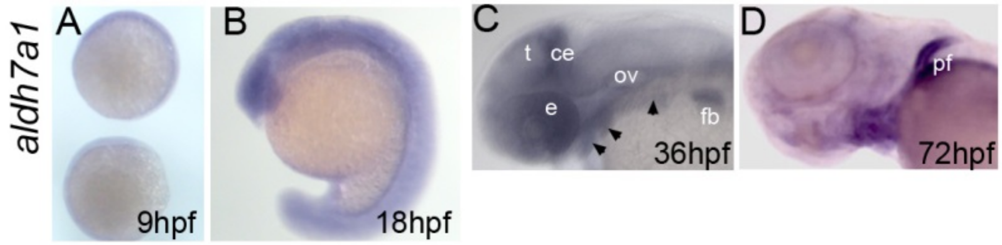

Figure S1. ***aldh7a1*** **expression.**
Whole-mount *in situ* hybridization of shows expression of *aldh7a1* at (A) 9hpf, (B) 18hpf,(C) 36hpf, and (D) 72hpf.e,eye; t, tectum; ce,cerebellum; ov, otic vesicle; fb, fin bud; pf, pectoral fin. Arrow head indicates pharyngeal arches.

Figure S2

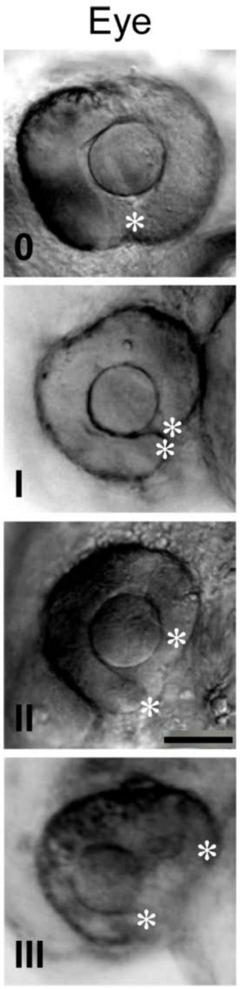

Figure S2. Grades of severity for eye development.

Classification of phenotype severity following morpholino injections. Four grades of coloboma (0, least severe; I, II,and III, most severe) classified by distance between edges of optic fissure at ~28 hpf, marked by white asterisks. Scale bar: 65 µm

Figure S3

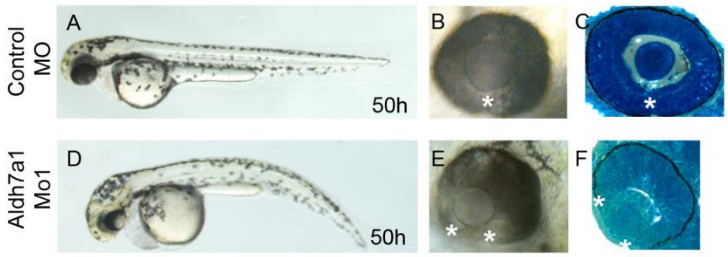

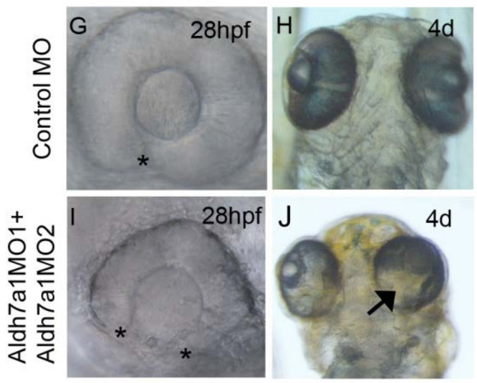

Figure S3. *aldh7a1* loss-of-function phenotype.
(A,D) Aldh7a1MO1 injected embryos developed bent tail compare to control MO injected embryos. (A-F) Lateral view of zebrafish eye at 50 hpf. Co-injection of Aldh7a1MO1 and Aldh7a1MO2 developed coloboma(I n=36/41,J n=25/31), compared to(G,H) control MO injected embryos.(C-F) Sagittal section of eye. Asterisks indicate edges of optic fissure, arrow indicates ventral defect in the eye at 4d.

Figure S4

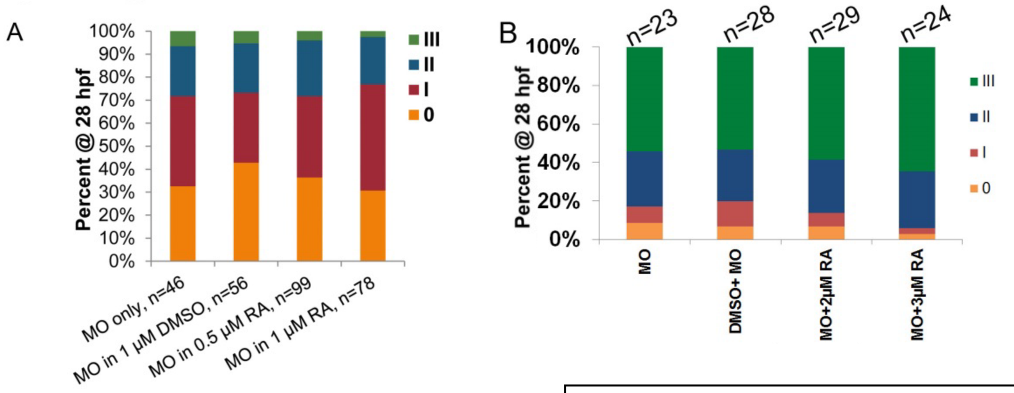

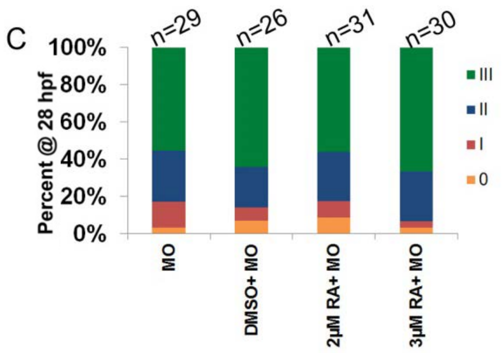

Figure S4. *aldh7a1* morphant phenotype is not rescued by retinoic acid .
(A-C) *aldh7a1* morphant embryos were incubated in retinoic acid (RA) beginning at; (A) 20-somite stage for 1 hour, (B) 2 -somite stage for 2 hours, and (C) 10-somite stage for 2 hours. The distribution of eye phenotypes (0, I, II, and III) was recorded at 28hpf. RA had no visible effect compared to DMSO control.

Figure S5

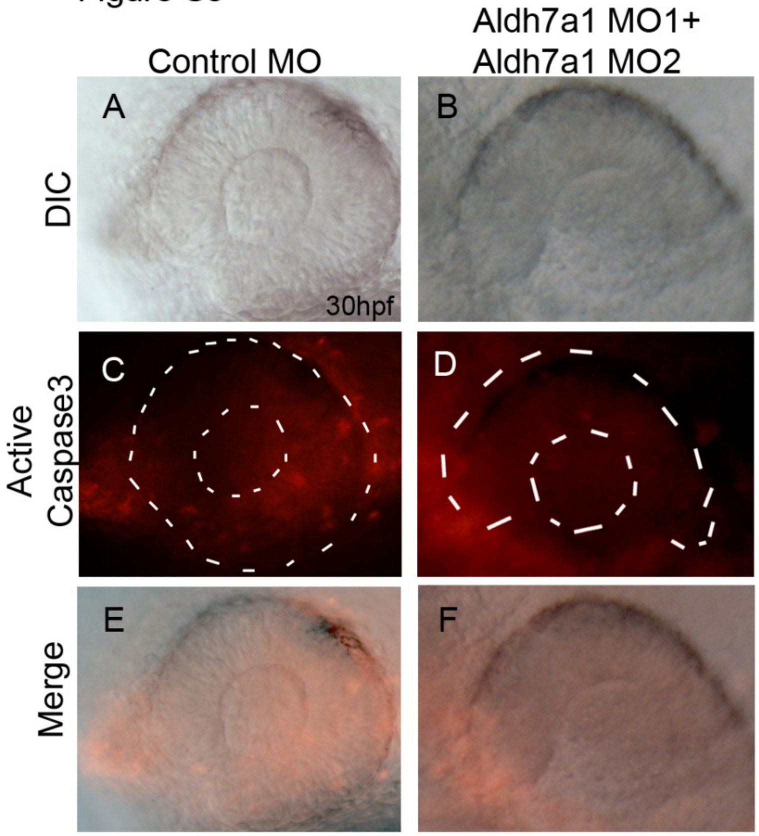

Figure S5. Coloboma in *aldh7a1*morphant fish is not due to apoptosis.

Active Caspase3 staining of control (C,E) and *aldh7a1* morphants embryos (D,F). White lines in C, D demarcates the eye.

Table S1:

Gene	cDNA IMAGE clone*	Primer†	5'→3' Sequence
aldh7a1	2600757	antisenseprobe_FWD	TGGTTGTACCGTCTGCTGTC
		antisenseprobe_REV	CAAAACAAGGGTTGCAATGA
		ORF_FWD	CGGAATTCCGATGTCGACTCTGCTTATTAACCAAC
		ORF_REV	CCCTCGAGGGGGCAACATCATGATTCATTCAA
nlz1	7405421	ORF_FWD	GATCCAGATGAGCGAACTGC
		ORF_REV	TAATCACTGGTACCCAAGAGCA
vax2	9038293	ORF_FWD	ATGTTTGATCAAGCCACGA
		ORF_REV	GGTAGAGGGGAGAAGATTACAGG

*IMAGE clones obtained from OpenBiosystems (www.openbiosystems.com); †primers obtained from BioServe (www.bioserve.com).

Table S1

List of Image clones and Oligonucleotides used in this study.
